# Reading Picture Books With Elements of Positive Psychology for Enhancing the Learning of English as a Second Language in Young Children

**DOI:** 10.3389/fpsyg.2019.02899

**Published:** 2020-01-23

**Authors:** Anna N. N. Hui, Bonnie Wing-Yin Chow, Eva S. M. Chan, Man-Tak Leung

**Affiliations:** ^1^Department of Social and Behavioural Sciences, College of Liberal Arts and Social Sciences, City University of Hong Kong, Kowloon, Hong Kong; ^2^Department of Chinese and Bilingual Studies, Faculty of Humanities, Hong Kong Polytechnic University, Kowloon, Hong Kong

**Keywords:** dialogic reading, positive psychology, receptive vocabulary, syntactic complexity, verbal creativity

## Abstract

This study aimed to investigate the learning effectiveness of reading picture books with EMPATHICS elements using dialogic reading techniques in enhancing young children’s English language learning and creativity. EMPATHICS is an acronym of Emotion and Empathy, Meaning and Motivation, Perseverance, Agency and Autonomy, Time, Habits of Mind, Intelligences, Character Strengths, and Self Factors (Oxford, 2016). It adopted a quasi-experimental design, and 78 kindergarten children aged from 4 to 5 years old in a cluster group were randomly assigned to the experimental and control groups. Both groups read the same four picture books with their homeroom teachers, including two readers suggested in the curriculum and two picture books with enriched elements for 12 sessions over 8 weeks. A doubly multivariate analysis was used to measure the main time and group effects and the interaction effect on the performance of English receptive vocabulary, syntactic complexity, and verbal creativity of the two groups across three different times. There were significant differences only in the interactive effect on syntactic complexity. Children in the experimental condition gave responses with more complex syntactic structures. Significant time effects for receptive vocabulary, syntactic complexity, and verbal creativity were observed in all children. Reading enriched English texts better prepares children to creatively and effectively express themselves. This study extends previous research in two ways. First, this study is one of the few studies on the effectiveness of dialogic reading using EMPATHICS-enriched picture books among young language learners. Second, this study investigates the effects of dialogic teaching on English as a second language development in young children. The educational implications will be discussed.

## Introduction

Reading story books to young children in an interactive way on a daily basis is an indicator of quality early childhood education (Siraj-Blatchford et al., 2008) and a significant predictor of language learning in primary education (van Druten-Frietman et al., 2016) and of language and literacy outcomes in longitudinal studies (Ulferts et al., 2019). Adult–child book reading is a kind of extended intervention technique that enhances children’s language skills for reading and written language outcomes when children actively engage in reading (Scarborough and Dobrich, 1994; Swanson et al., 2011). Teachers may use dialogic reading techniques, such as questioning, elaborating information, and initiating discussions, to encourage verbal interaction with children (Zevenbergen et al., 2003; Swanson et al., 2011) and to actively engage children in learning (Seligman et al., 2009).

In dialogic reading, children become a storyteller during book reading. Adults, on the other hand, become an active listener, audience, and questioner. Adults provide appropriate assistance to the child by using an evocative approach when reading a story, such as asking the child about the picture or content of the story, supporting the child in telling the story along with adults (e.g., Haden et al., 1996; Whitehurst and Lonigan, 1998; Swanson et al., 2011; Flack et al., 2018). There are significantly greater language gains than when adults simply read a book to children (Arnold and Whitehurst, 1994; Hargrave and Sénéchal, 2000; Chow and McBride-Chang, 2003). Additionally, children enjoy dialogic reading more than traditional teaching because the addition of prompts during reading together can enable adults to follow children’s interests and attention (Valdez-Menchaca and Whitehurst, 1992). The pleasure of reading is beneficial to the child’s language development (Zevenbergen et al., 2003). Recent studies found that dialogic reading has a positive effect on language and literacy development for the child, mainly in encouraging receptive vocabulary development and interest in reading, for both Chinese (Chow et al., 2008, 2010), and English language learning in elementary classrooms (Chow et al., 2017). Young children in Hong Kong received formal education in reading and writing, in both Chinese and English languages, at age 3 in Kindergarten, as stipulated in the Kindergarten Education Curriculum Guide (Education Bureau, 2017).

### Effects of Dialogic Reading

Swanson et al. (2011) conducted a meta-analysis of reading interventions for children at risk for reading difficulties from preschool to third grade. The dialogic reading approach is one of the frequently used interventions, and outcome measures are mostly expressive vocabulary, receptive vocabulary, word recognition, and semantic complexity (e.g., number of nouns, verbs, modifiers); and of the 27 studies, only one or two studies used syntactic complexity (e.g., mean length of utterance). These authors concluded that extended child–adult dialog and questioning around storybooks can improve literacy outcomes, mainly receptive and expressive vocabulary although fewer studies use syntactic variable.

Verbal creativity is another language outcome examined in studies with young children, schoolchildren, and college students. Smogorzewska (2014) found that both reading stories to young children, and asking them to make stories enhanced creativity as measured by semantic structure (continuous episodes), narration cohesion (time sequence, cause–effect), story complexity (number of connections among characters), story length (number of words), and originality (novel elements). Aerila and Rönkkö (2015) integrated arts, story reading, and telling as a creative learning process. They first read part of a story to a group of young children and they further verbally elaborated the story and made art craft of the characters and episode, then they read about the original ending, their new elaboration, and shared collaboratively about their own artistic products. Creative outcomes included verbal creativity as measured by the new story, and visual creativity as presented by hand drawn pictures and handmade characters.

Flack et al. (2018) conducted another meta-analysis on how children’s language acquisition is related to reading picture/story books using 38 studies with 2,455 children. Children learned 3.025 words (raw change) on average, and 46% of the words were included in the reading process. Dialogic reading techniques increase word learning in children by at least one word when adults describe pictures and ask questions during reading, regardless of whether teachers or parents or caregivers serve as the reader. These authors also suggested some new future directions for research. Children aged between 3 and 5 years who are read the same storybooks repeatedly learned more words. Children between 2 and 10 years could learn approximately two to five new words (approximately 2.77 nouns, 3.10 verbs). Reading styles, exposure to the same storybooks, and learning nouns and verbs are all significant moderators when studying the dialogic reading approach with young children.

Of the 38 studies in Flack et al.’s (2018) meta-analysis, four major types of reading stimuli were employed. First, two studies used a wordless picture book (Ard and Beverly, 2004; Abel and Schuele, 2014). Second, approximately 15 studies used author-created picture books embedded with targeted words that were mainly nouns (e.g., Blewitt et al., 2009; Houston-Price et al., 2014; Flack and Horst, 2018). Third, two studies used adapted picture books with target words or grammar structures (McLeod and McDade, 2011; Evans and Saint-Aubin, 2013). The final type of picture books were popular story books in children’s literature with no specific target words or structures (Beck and McKeown, 2007; Pullen et al., 2010; Strasser et al., 2013; Chen and Liu, 2014). Commercial picture books differ from wordless, author-created or adapted children’s picture books in that the former have significantly more words, more types of words, and more grammar structures. Flack et al. (2018) did not define or observe that type of picture book was a significant moderator. The dialogic reading technique had the greatest effect.

Reading stories with themes on resilience, or enriched with sensory stimuli also enhance creativity among older students. Boytos et al. (2015) discovered that college students scored higher in the Torrance Tests of Creative Thinking after reading an underdog story than those who read a top dog. The underdog displayed resilience when undergoing hardship in life and creativity in problem solving. Bos et al. (2015) found that kindergarten children with higher sensory richness scores wrote highly original stories with more sensory words and situational words. Webb and Rule (2014) integrated story reading with humor, wisdom, and emotion when learning about health and nutrition in a second grade classroom. Students displayed higher creativity in figural creativity but reported no significant differences on enjoyment of book, enjoyment of making figural transformation, and perceived creativity.

In this study, award-winning commercial picture books written in English for children aged between 3 and 6 years were chosen for three reasons: the narrative level was appropriate for the kindergarten age group, they had appealing illustrations to children, and they had enriched elements of the EMPATHICS model (Oxford, 2016) outlining the psychological dimensions of positive language learning. EMPATHICS is an acronym for Emotion and Empathy, Meaning and Motivation, Perseverance, including Resilience, Agency and Autonomy, Time, Habits of Mind, Intelligences, Character Strengths, and Self Factors, especially self-efficacy. The enriched content provides more interesting elements to enhance vocabulary and syntax compared with traditional reading, which usually focuses on repetitive semantic (e.g., food items) and syntactic elements (e.g., “I like …”). To our knowledge, this study is the first study to adopt the EMPATHICS model for studying how beneficial it is for young children’s L2 learning. The current study aimed to investigate the effectiveness of reading picture books with EMPATHICS elements using dialogic reading techniques for enhancing young children’s English language development and verbal creativity.

## Materials and Methods

### Participants

The study was a quasi-experimental design with three different time points, including a pretest, mid-test, and posttest, to investigate how picture books with EMPHATICS elements and read using dialogic reading techniques help the language and creativity development of young children learning English as a second language. Ethical approval was obtained from the Ethics and Research Committee of the university. Parents provided written and informed consent for their child and themselves to take part in the study. Seventy-eight kindergarten children aged from 4 to 5 years in cluster groups were randomly assigned to the experimental and control groups. Initially, a total of 89 kindergarten children in three K2 classes and three K3 classes (43 in the experimental condition and 46 in the control condition) were included. Only 39 children (19 boys and 20 girls) in the experimental group and 39 (18 boys and 21 girls) in the control group completed the three assessments, yielding an attrition rate of 12.3%, usually due to sick or casual leave during the second or third assessment times.

### Procedure

At the baseline measurement, each child was individually tested for approximately 15–20 min in the kindergarten classroom by trained psychology undergraduate and postgraduate students. With a double-blind design, the experimenters and the child participants did not know which children belonged to the experimental or control groups. Children were briefed that they would use English to play all games before the tests. There were three tests for the children: an English receptive vocabulary test, a story-telling task (STT), and free conversation. After all the tests, a cartoon sticker was given to the child as a token of appreciation, and each child was given an English picture book to bring home as another token.

Four classes of kindergarten children from two different age groups (ages 4 and 5) were randomly assigned into two groups: (a) an experimental group with dialogic reading first with ordinary English stories and then with creative English stories or (b) a control group with traditional reading first with ordinary English stories and then with creative English stories. After the baseline measurement, the former 4-week dialogic reading sessions with typical English reading as assigned by the kindergarten were conducted in the experimental group, whereas traditional reading with the same typical English reader was implemented in the control group. There were a total of 12 lessons (240 min) and 20 min for each lesson. Two participating teachers in the dialogic reading group were trained by the researchers in how to use dialogic reading techniques to link creativity and vocabulary learning in the story books. Teaching materials with clear instruction and procedures, i.e., using the Prompt–Evaluate–Expand–Repeat sequence and five types of questions with CROWD as the acronym, namely, Completion, Recall, Open-ended, Wh-words, Distancing, were provided for teachers (please see Appendix I for sample questions). There was a classroom observation in the first or second lesson taught by trained teachers to ensure fidelity of implementation. According to a recent review on fidelity of dialogic reading studies in early childhood education of Towson et al. (2019), the fidelity level of the current study can be classified as the highest level when “authors stated training was provided and gave a detailed description of training” (p. 136).

The classes were also recorded to evaluate the whole teaching process. After the 4-week intervention, a mid-test assessment was conducted with the same procedures and assessment materials as those completed at the baseline assessment. The next 4-week dialogic reading sessions with English stories with EMPATHICS elements were implemented in the dialogic group, and four sessions of traditional reading with the same English stories with enriched elements were conducted in the control group. The posttest measurement was completed within 1 month of the reading intervention taking place.

To control for cognitive ability and parental influence, children’s non-verbal reasoning ability was assessed by Raven’s standard progressive matrices (Raven et al., 1996), and no significant difference was found (*t* = 0.854, *p* = 0.397) between the experimental and control groups. The Parent–Child Interaction Questionnaire (Yau and Yang, 2014), and Chinese Early Parental Involvement Scale (Lau et al., 2012) were used to measure psychological and language interaction, and parental involvement in school. No significant differences in parent–child interaction overall mean scores (*t* = 0.653, *p* = 0.516), and those of parental involvement (*t* = 0.487, *p* = 0.628) were found in between the experimental and control groups.

### Materials

Four English story books were used for the 8 weeks of the 12 sessions of dialogic reading lessons, including two typical English readers assigned by the school used in the first six sessions and another two English EMPATHICS picture books in the second six sessions. Features of these readers and picture books are described in great detail in Table 1.

**TABLE 1:** Features of the English readers and picture books.

Title	Author	Year	Publisher	Features
Typical Readers ⟨⟨Farm Animals⟩⟩	Anoynmous	2003	Crystal Education Publication	Repeated sentence structure: I see … Vocabulary on farm animals, e.g., cow, pig, chicken, and etc. No elements on EMPATHICS
⟨⟨I like…⟩⟩	Jillian Cutting		Sunshine Books	Repeated sentence structure: I like eating … Vocabulary on food items, e.g., ice-cream, burgers, spaghetti, and etc. Few elements on EMPATHICS Emotion: favorite food
EMPATHICS Picture Books ⟨⟨Along a long road⟩⟩	Frank Viva	2011	Little, Brown Books for Young Readers	Little repetition of sentence structure Vocabulary on adverbs of position, e.g., on, over, under, and etc. Emotion: Relaxing when cycling along a long road across the city Meaning and Motivation; Perseverance: including resilience and hope; Agency: the cyclist’s autonomy to travel along the long road by bike; Time: temporal appraisal of time passing spontaneously as the cyclist passes along the long road; Habits of mind: gathering data through all senses as the cyclist sees, and hears things along the long road; Intelligences: bodily kinestics, intrapersonal, interpersonal, logical, visual–spatial, verbal; Character strengths: curiosity and perseverance in discovering about the city; Self factors: self-efficacy
⟨⟨Grandpa Green⟩⟩	Lane Smith	2011	Roaring Brook Press	Little repetition of sentence structure Vocabulary on life events, e.g., boyhood, wedding, baby, and etc. Emotion: Acceptance as Grandpa grows across the lifespan Meaning: lifespan development of Grandpa from childhood, adolescence, adulthood, older adulthood Perseverance: surviving through the World War II Agency: service in the army and for the family in raising the family Time: time perspectives of past, present and future when listening to grandpa’s life story Habits of mind: listening with understanding and empathy, finding humor in grandpa Intelligences: bodily kinestics, intrapersonal, interpersonal, logical, visual-spatial, verbal Character strengths: creative, Self factors: self-efficacy in horticultural arts

### Instruments

#### Receptive Vocabulary in English

##### The Peabody Picture Vocabulary Test IV (PPVT-IV; Dunn and Dunn, 2007)

This test is an English-graded vocabulary test for children aged between 3 and 6. Children were orally presented a vocabulary item and asked to choose the picture for this item out of a four-picture grid. The four-picture grid included pictures representing a target word, an onset distractor, a rhyming distractor, and an unrelated distractor. For example, in one testing trial, “Cat” was a target word, “Hat” was a rhyming distractor, “Cookie” as an onset distractor, and “Dog” was an unrelated distractor. The Cronbach’s alpha was 0.982 for Time 1. The correlation coefficients between Time 1 and the other two time points were Time 2 (*r* = 0.679, *p* = 0.000) and Time 3 (*r* = 0.722, *p* = 0.000), and between Time 2 and Time 3 (*r* = 0.741, *p* = 0.000), indicating satisfactory test and retest reliability.

#### Syntactic Complexity in English

##### Edmonton Narrative Norms Instrument (ENNI; Schneider et al., 2002; Schneider and Hayward, 2010)

The story-telling test measures children’s syntactic development. In the test, a series of five wordless pictures was shown to participants in an individual session. Each participant was required to tell a story about the pictures on his/her own in English. Before telling the story, the participants were allowed 1 min to look through all pictures, and they were asked and briefed to tell an interesting story later. Five-picture stories in A1 and five-picture stories in B1 were used. In the pilot test, half of the children randomly used the A1 story, and another half used the B1 story. In the baseline measurement and posttest assessment tasks, the B1 story was used while the A1 story was used in the pretest to reduce the effect of time. The Cronbach’s alpha value for the following 10 items for Time 1 was 0.709, indicating satisfactory reliability. An overall mean score was computed using the 10 items. All stories were video-recorded and scored in the target items: (a) the amount of words - total number of words (TNW), (b) number of different words (NDW), (c) mean length of communication units (MLCU), (d) included utterance (IU), (e) independent clause (IC), (f) dependent clause (DC), (g) clausal units (CU), (h) complexity index [CI = (IC + DC)/IC], (i) story grammar (SG) units to evaluate overall content and marco structure (organization) for characterizing good stories, and (j) first mentions (FM) to measure the referential cohesion using FM of characters and objects first when telling a story. The correlation coefficients between Time 1 and the other two time points were Time 2 (*r* = 0.386, *p* = 0.000) and Time 3 (*r* = 0.470, *p* = 0.000), and between Time 2 and Time 3 (*r* = 0.526, *p* = 0.000), indicating moderate test–retest reliability.

#### Verbal Creativity in English

##### Story-telling task (STT; Hennessey and Amabile, 1988; Hui et al., 2013)

The STT was conducted by an experienced researcher and trained research assistants. Each child was presented with an unseen picture and was asked to tell a story about the picture. In this test, child participants were provided 3 min for preview and 5 min to create their story. The participants were allowed to continue until they indicated completion. The storytelling process was digitally recorded and then independently evaluated by two raters for 13 criteria: (1) relevancy to the story, (2) ability to describe the story, (3) ability to organize the story, (4) ability to express, (5) ability to show emotions, (6) ability to speak in an audible tone, (7) ability to add conversations, (8) ability to include humorous elements, (9) ability to include creative elements, (10) ability to identify problems and find relevant solutions, (11) ability to name the story, (12) ability to make story by themselves, and (13) ability to use vocabulary. Each criterion was rated on a five-point scale (from 0, lowest, to 4, highest). A composite score was calculated for each participant. Each story was rated by two trained researchers. There were positive correlations between the composite scores calculated by the two markers for the three tests (*r* = 0.56 ∼0.73, *p* < 0.001), indicating moderate interrater reliability. The correlation coefficients between Time 1 and the other two time points were Time 2 (*r* = 0.439, *p* = 0.000) and Time 3 (*r* = 0.468, *p* = 0.000), and between Time 2 and Time 3 (*r* = 0.545, *p* = 0.000), indicating moderate test–retest reliability.

## Results

Descriptive statistics of the vocabulary, creativity, and syntax scores across three time points are listed in Table 2. An independent sample *T*-test was conducted to examine whether there was any initial difference among the pretest scores and no significant differences were found: receptive vocabulary (*t* = -0.325, *p* = 0.746), verbal creativity (*t* = 1.182, *p* = 0.241), and syntactic complexity (*t* = 1.334, *p* = 0.186).

**TABLE 2:** Means and standard deviations of variables.

	Time 1		Time 2		Time 3	
	*M*	*SD*	*M*	*SD*	*M*	*SD*
**Experimental group (*N* = 39)**						
Receptive vocabulary	26.53	8.04	27.42	7.87	30.79	9.09
Syntactic complexity	4.84	3.55	7.39	4.12	8.74	4.33
Verbal creativity	14.01	4.48	16.34	4.02	20.47	6.04
**Control group (*N* = 39)**						
Receptive vocabulary	26.79	8.44	27.82	8.39	30.13	7.93
Syntactic complexity	3.31	2.75	4.61	3.30	5.38	2.92
Verbal creativity	12.33	4.89	13.91	5.03	18.16	4.51

A two-way (two groups × three times) repeated measured MANOVA was conducted to assess whether there were differences across the three time points and between the DR and TR group. The assumption of sphericity was not violated and thus the sphericity assumed values were used. Statistically significant multivariate effects were found for the main effects of group, Wilks’ lambda = 0.781, *F*(3,74) = 6.909, *p* < 0.001, eta^2^ = 0.219, and time, Wilks’ lambda = 0.340, *F*(6,71) = 22.953, *p* < 0.001, eta^2^ = 0.66, but no overall interaction effect between time and group, Wilks’ lambda = 0.911, *F*(6,71) = 1.151, *p* = 0.343.

Within the same group, the time effect showed the difference between reading with typical reader (Time 2) and the enriched EMPHATHICS picture books (Time 3). There was a significant group effect, Wilks’ lambda = 0.781, *F*(3,74) = 6.909, *p* < 0.001, eta^2^ = 0.219, indicating the difference between the dialogic reading group and the traditional reading group when using the same type of reading materials at the same period. A follow-up ANOVAs revealed that the statistically significant change from Time 1 to Time 3 was only for the syntactic complexity variable, *F*(1,76) = 49.956, *p* < 0.001, eta^2^ = 0.397, and that the change was different from the two groups, *F*(1,76) = 4.676, *p* < 0.05, eta^2^ = 0.058. The effect size was small. Table 3 shows the time and group and interaction effects of the variables.

**TABLE 3:** Effects of time and group on variables.

	*F*	Sig.	η^2^
**Within group (time effect)**			
Receptive vocabulary	31.455^∗∗∗^	0.000	0.293
Verbal creativity	101.95^∗∗∗^	0.000	0.573
Syntactic complexity	49.96^∗∗∗^	0.000	0.397
**Between group (group effect)**			
Receptive vocabulary	0.005	0.944	0.000
Verbal creativity	6.184*	0.015	0.075
Syntactic complexity	16.94^∗∗∗^	0.000	0.182
**Interaction effect (time × group)**			
Receptive vocabulary	0.419	0.519	0.005
Verbal creativity	0.390	0.534	0.005
Syntactic complexity	4.676*	0.034	0.058

*p < 0.05, ^∗∗∗^*p* < 0.001.

**FIGURE 1:**
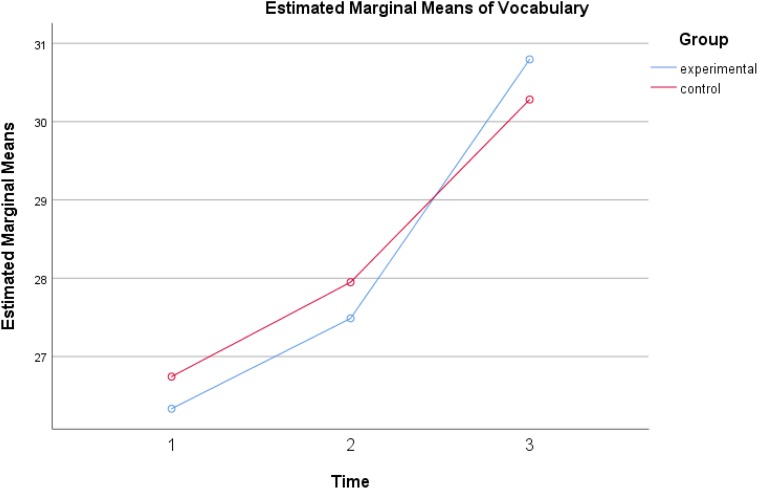
Receptive vocabulary gains of experimental group and control group across three times.

**FIGURE 2:**
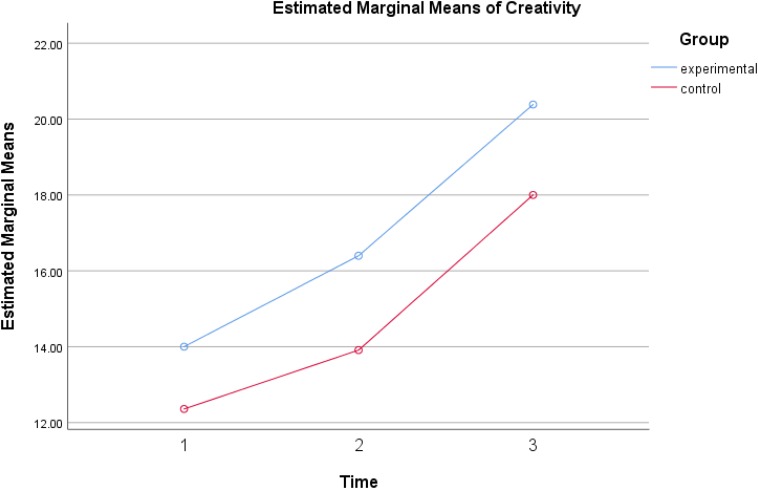
Verbal creativity gains of experimental group and control group across three times.

**FIGURE 3:**
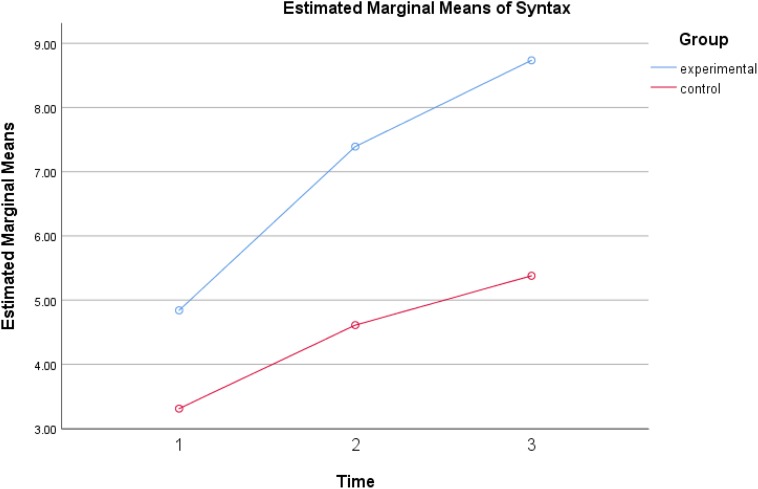
Syntactic complexity gains of experimental group and control group across three times.

Follow-up ANOVAs show that the means of language and creativity scores suggest that all children had significantly higher across the three time points in receptive vocabulary, *F*(2,152) = 16.379, *p* < 0.001, eta^2^ = 0.177, syntactic complexity, *F*(2,152) = 57.95, *p* < 0.001, eta^2^ = 0.433, and verbal creativity, *F*(2,152) = 23.597, *p* < 0.001, eta^2^ = 0.237. Figures 1–3 show the increases of the outcomes across the three time points in all participants. All children benefited from reading both typical reader and the enriched picture books; however, more observable gains were found when both groups reading the enriched picture books.

Significant differences were found between the experimental group and the control group in verbal creativity, *F*(1,76) = 6.184, *p* < 0.05, eta^2^ = 0.075 and also in syntactic complexity, *F*(1,76) = 16.94, *p* < 0.001, eta^2^ = 0.182 but no such difference was reported in vocabulary, *F*(1,76) = 0.005, *p* = 0.944. Inspection of the Figures 2, 3 suggested that the dialogic reading group told stories with higher syntactic complexity, and higher creativity than the control group. Both groups gained similarly in receptive vocabulary and thus no significant differences were observed among them.

## Discussion

This study investigated the learning effectiveness of reading picture books with EMPATHICS elements (Oxford, 2016) using dialogic reading techniques in enhancing young children’s English literacy development as a second language. The findings have showed both groups reported similar pattern of vocabulary, syntactic, and creativity development when reading frequently with teachers after the 12-week intervention. More advanced developments in syntax and creativity are reported when using dialogic reading techniques of dialogic teaching. This study extends previous research by investigating the effectiveness of dialogic reading techniques on English as second language development among kindergarten children using typical school readers and stories enriched with positive psychology elements.

### English Receptive Vocabulary

The increase in young children’s English vocabulary across the 12 weeks has indeed indicated that reading picture books to children in early childhood classroom frequently brings benefits to their receptive vocabulary, regardless of using traditional or dialogic techniques. These findings are partly consistent with the literature that picture book reading strengthens students’ vocabulary knowledge (Flack et al., 2018). Teachers reading to children in a daily basis is an important indicator of quality early childhood environment (Siraj-Blatchford et al., 2008), and serving as a predictor of children’s later language outcomes (van Druten-Frietman et al., 2016; Ulferts et al., 2019). The impact of using EMPATHICS enriched storybooks on vocabulary gain is reported when these books are used between Time 2 and Time 3. This finding is consistent with a recent study on reading extensively with different types of books to children in a dialogic method can enhance expressive vocabulary (Wesseling et al., 2017).

The lack of significant positive effects of dialogic reading on English vocabulary knowledge may be due to the relatively short implementation period. With a longer implementation time, dialogic reading approach which promotes more interactions might have a beneficial effect on language skills like other interventions of interactions on the first language acquisition, such as extratextual interactions programs of over 30 weeks (Wasik et al., 2006). In some successful studies, vocabulary items related to the picture books have been included as assessment items and expressive vocabulary can serve as alternative vocabulary outcomes (Ong, 2017).

### English Syntactic Complexity

Our findings have indicated that reading frequently to children with different types of stories promotes syntactic development in L2 English in young learners, in addition to vocabulary enhancement. This finding is consistent with previous studies (Smogorzewska, 2014; Flack et al., 2018). The type of reader does make a difference to literacy development, and does the reading approach as well. The original books chosen by the kindergarten teachers are written and produced by textbook suppliers. These picture books often focus on one or two repetitive sentence patterns, related vocabulary, and simple illustrations with an intention to use repeated reading and rote learning to foster semantic and syntactic gains. Language acquisition in L2 learners follows a similar developmental theory of an integration of complex syntax, lexical learning, and vocabulary development as suggested by Dye et al. (2019). Story reading is also a good pedagogical strategy to foster syntactic development (Schneider and Hayward, 2010).

The EMPATHICS model enables teachers to take into account the psychological aspects of the language learner. The positive impact of EMPATHICS elements is manifested when enriched storybooks are read between Time 2 and Time 3. To sustain children’s reading motivation and support children to be fluent readers, the EMPATHICS elements serve as excellent criteria for teachers and parents to choose books that strengthen both learners’ reading behaviors but also agency, autonomy, and self-efficacy of children (MacIntyre, 2016; Oxford, 2016).

Both incorporating extended child–adult dialog by questioning around the stories, and using traditional reading approach encourage children to speak longer and complex sentences, communicate more eagerly, and express themselves more willingly. This finding is consistent with Lonigan and Whitehurst’s (1998) study that children produced more lengthy sentences with more different words when reading unfamiliar book with dialogic reading approach. The extended child–adult dialog and questioning techniques with CROWD can help young children read the text creatively and extensively, and encourage them to read with greater intrinsic motivation (Walsh and Blewitt, 2006; Chow et al., 2017).

### English Verbal Creativity

Although the increase in young children’s verbal creativity in the two groups did not show a significant interaction effect, their verbal creativity increases when reading constantly with both types of readers across the 12 sessions. Children tend to tell more creative stories when they are asked frequently about questions generated from the readers. The EMPATHICS enriched readers serve as interesting stimuli to enhance creativity through cultivating children’s imagination (Kohm et al., 2016; Moedt and Holmes, 2018). Kohm et al. (2016) reported teachers observed children actively creating “new adventures based upon the story’s content and language” and engaging in more social play with peers and generating positive affect.

Previous studies have also found similar mixed results on older children and in children from other non-Chinese cultures (Fleith et al., 2002; Hommel et al., 2011; Leikin and Tovli, 2014). Leikin and Tovli (2014) examined the creative performance of two groups of kindergarten children under 6 years old who spoke both Russian and Hebrew or only Hebrew. Verbal creativity was assessed by asking children to generate as many responses as possible to three semantic categories (animals, food and things to be taken on a picnic) and generating as many solutions as possible to a problem solving task. Bilingual children outperformed monolingual children in semantic tasks but did not show significant differences in the problem solving task. Fleith et al. (2002) found no significant difference in figural creativity among Grade 5 schoolchildren, half of whom spoke both Brazilian and English, and half of whom were monolingual children, after participating in a 15-week creativity training program. Hommel et al. (2011) studied English vocabulary and two cognitive tests, including a remote associates task and an alternate uses task between two groups of college students who were highly proficient Dutch–English bilingual students living in the Netherlands and low proficiency bilingual students with German origins. The highly proficient group of Dutch–English participants scored higher on English vocabulary and the remote associates task, but the low proficiency group scored higher in fluency and the alternate uses task.

Creativity among young children may vary with the nature of the creativity tasks. Familiar tasks tend to generate more creative responses, e.g., creative artwork (Fleith et al., 2002; Webb and Rule, 2014; Aerila and Rönkkö, 2015). The STT is a familiar activity in early childhood classroom when compared with the remote associates tasks or alternate use tasks (Hommel et al., 2011) or instance tasks (Leikin and Tovli, 2014). In other studies with older students, language proficiency is found to be an important variable in studies showing an advantage in creativity. Given the young age of participants in the current study, their language proficiency is still developing and of similar level, and it is reasonable to observe that reading both types of readers have enhanced verbal creativity. The lack of significant positive effects of dialogic reading on verbal creativity can be due to the emerging language proficiency in L2 when learning two languages in early childhood (Kharkhurin, 2018).

### Limitation and Further Studies

There are three major limitations in this study. First, the dialogic reading approach is implemented for only 12 sessions in this study. Further studies can involve a longer implementation period to provide a clearer picture of its effects on students’ language development. Second, the sample size is small which might affect the effect size. Third, this study has demonstrated positive effects of EMPATHIICS-enriched literacy texts on English sentence complexity but does not examine the underlying mechanisms. Future research can include observation of teacher–child interactions to examine factors mediating these causal links. Variables of the children’s well-being may also be included and a larger sample may be used to improve the effect size.

## Conclusion and Implications

This study has provided evidence for the effectiveness of EMPATHICS-enriched reading on facilitating language development through enhancing syntactic development in young children learning Chinese as a first language and English as a second language. The findings have several major implications for language learning and second language learning. First, the present study has extended the application of positive psychology in language learning and early childhood classroom by demonstrating close links between the EMPATHICS model and English language learning in kindergarten, in addition to direct instruction, on positive psychology as investigated by Kristjánsson (2012) and Shoshani and Slone (2017). This finding stimulates further research in this area, particularly in investigating the underlying mechanisms of these phenomena. Additionally, this study has provided a new direction for educators and parents to design and implement learning activities that enhance positive emotions, character strengths, and joy in learning. It is feasible to integrate positive psychology in language education (Ciarrochi et al., 2016). The findings have demonstrated the importance of providing a stimulating learning environment in language instruction for young children in kindergarten. Future directions include a longitudinal study of how young children further develop their language skills in both Chinese and English from kindergarten to primary school.

## Data Availability Statement

The datasets generated for this study are available on request to the corresponding author.

## Ethics Statement

The studies involving human participants were reviewed and approved by City University of Hong Kong. Written informed consent to participate in this study was provided by the participants’ legal guardian/next of kin.

## Author Contributions

AH served as the principal investigator of the research project described in the manuscript. BC, EC, and M-TL served as co-investigators. All authors conducted the study together, visited the experimental and control classrooms, trained the teachers in the experimental group, and discussed about data analyses.

## Conflict of Interest

The authors declare that the research was conducted in the absence of any commercial or financial relationships that could be construed as a potential conflict of interest.

## Appendix I

### Dialogic Reading

a) Steps in dialogic reading:

• Prompt—Prompt children to speak by questioning five different types of questions (CROWD):
• Evaluation—Evaluate children’s answers
• Expansion—Expand children’s answers into complete sentences
• Repetition—Let the children repeat the expanded sentences.

Questions used to prompt children to speak:

1. Completion: Leave the end of a sentence blank for students to fill in
2. Recall: Let students recall some previous content of a story
3. Open-ended: Encourage students to describe pictures in the story book using their own words, and do not have a definite answer
4. Wh-question: Who, What, Where, When, Why, and How
5. Distancing: Relate the content in the story book to daily life of students, ask about their personal experience or feelings, and do not have a definite answer.

School reader:

Book Name: I Like…        Story by: Jillian Cutting

**Table:**

	Question	Suggested answer	Prompt	Evaluation–expansion–repetition
1	Do you remember what the little boy likes in the story?	Chicken, hamburger, milkshake, apple, ice-cream, pizza	Recall	Yes, he likes chicken. Let’s say it together “He likes chicken.”
2	Do you know what is ice-cream made of?	Milk, ice, sugar, cream	Wh-question	Correct. Ice-cream is made of milk, ice, sugar and cream. Please repeat “Ice-cream …”
3	Where do apples come from?	Apple tree	Wh-question	Right. Apples grow on apple trees.
4	What is/are the food(s) that both you and the little boy like?	Any possible answer	Distancing	Both of you like eating hamburger.
5	What the little boy doesn’t like/dislike in the story?	Spinach	Recall	Yes, he doesn’t like spinach.
6	Do you like spinach or any other vegetables?	Any possible answer	Distancing	Good. You like tomato.
7	What other foods do you like? Why?	Any possible answer	Distancing	Spaghetti. You like spaghetti because it is yummy.
8	What foods do you like the best/the most? Why?	Any possible answer	Distancing	You like cheese most because it tastes good.
9	What foods do you not like/dislike? Why?	Any possible answer	Distancing	You don’t like fish because it has bones.
10	What else do you think the little boy in the story will like?	Any possible answer	Open-ended	Yes, he will like cheese sandwiches.

Reading with EMPATHICS enriched picture book:

Book Name: Along a Long Road        Author and Illustrator: Frank Viva

**Table:**

	Question	Suggested answer	Prompt	Evaluation–expansion–repetition
1	Can you ride bicycle?	Any possible answer	Distancing	Yes, I can ride a tricycle. Let’s repeat.
2	Where did the boy in the book go to when riding his bicycle?	A small town, a circus	Recall	Yes he went to a small town on his bike.
3	Describe a place you saw in the book that the boy rode by.	In a tunnel, over a bridge, a grocery shop, and etc.	Recall	He went in and through a tunnel.
4	What did he hear when he went near a circus?	Any possible answer	Distancing	When he went near a circus, he heard children laughing.
5	The boy in the book forgot to take precautions when riding a bicycle, what has he forgotten to do?	Wear a helmet	Recall	Yes, for his safety, he has to put on a helmet.
6	Why should we wear a helmet when we ride a bicycle?	For protection	Open-ended	
7	How do you feel about riding bicycles on the streets in Hong Kong?	Teacher may want to talk about countries are more cyclist-friendly (Denmark)	Open-ended	It is too crowded to ride bicycles in HK because there are a lot of cars. In Denmark, people go to work by bike.
8	Why did the cyclist suddenly stop on the road?	He hit a bump/apple	Wh-question	Yes, he stopped when he hit a bump.
9	Why did the boy start all over again?	He likes riding on bicycle.	Open-ended	Yes, he likes cycling and enjoys riding on it again and again.
10	The cyclist rode on a _____ road.	Long	Completion	He rode along a long road.
